# Early experience of universal health coverage in Turkey on access to health services for the poor: regression kink design analysis

**DOI:** 10.7189/jogh.08.020412

**Published:** 2018-12

**Authors:** Abdullah Tirgil, Ipek Gurol-Urganci, Rifat Atun

**Affiliations:** 1 Department of Economics, College of Social Sciences and Humanities, Northeastern University, Boston, Massachusetts, USA; 2 Department of Public Finance, Faculty of Political Sciences, Ankara Yildirim Beyazit University, Ankara, Turkey; 3 London School of Hygiene and Tropical Medicine, London, UK; 4 Harvard T.H. Chan School of Public Health, and Harvard Medical School, Harvard University, Boston, Massachusetts, USA

## Abstract

**Background:**

In 2003, the Turkish government introduced a major health system reform, the Health Transformation Program (HTP), aimed at achieving Universal Health Coverage (UHC). HTP has helped to expand insurance coverage and health benefits for the uninsured population groups, which included low-income households and the unemployed, through the Green Card scheme, a non-contributory health insurance funded by the government. The Green Card scheme expansion began in 2005 and increased rapidly after 2008, following the introduction of a new comprehensive benefits package, to cover an additional 13 million people.

**Methods:**

We examine the impact of the Green Card scheme on the utilization of outpatient, inpatient, specialist, and diagnostics services using the Turkish Health Survey data (2010), using a kinked regression design. We take advantage of a sharp break in the availability of health insurance at a particular income level (minimum wage) to examine the impact of the Green Card scheme on health service utilization.

**Results:**

Our results show that having a Green Card increases the fraction of people using outpatient services by 68.30 percentage points, inpatient visit by 34.60 percentage points, and specialist visit by 74.10 percentage points.

**Conclusions:**

Our findings suggest that a non-contributory health insurance program, such as the Green Card scheme in Turkey, could provide increased access to health care services by the poor and provide important lessons for countries which aim to introduce health programs targeting poor as part of a transition to UHC.

Evidence from cross country studies suggest that broader health coverage and pooled financing help to expand access to necessary care, with improvements in population health, particularly for poor people [1]. Several middle-income countries such as Turkey [2], Brazil [3,4], Mexico [3,4], and Thailand [5] have sought to achieve Universal Health Coverage (UHC), along with 30 other low-and middle-income countries [6] including China [7], Indonesia [6], Philippines [6] and South Africa [6].

Providing UHC is challenging in countries that have fiscal constraints. There is a strong pro-poor intent with UHC expansion, as was the case with Seguro Popular in Mexico, where the poor did benefit [8]. Paradoxically, however, in some cases when health care services are expanded as part of UHC the poor are often the last to benefit [9-11].

Gruber and others investigated the effect of extending insurance coverage to previously uninsured populations in Thailand. They also examined increasing reimbursement for the publicly-insured poor with respect to utilization of health services. They found that for the previously uninsured, the ‘30 Baht Scheme’ led to increased health care utilization in general, but a larger increase was observed for the poor who were previously publicly-insured [12].

Beginning in 2003, the Republic of Turkey introduced the Health Transformation Program (HTP), a comprehensive health system reform aimed at UHC, in order to provide financial risk protection to all citizens and to overcome major inequities in health outcomes. The HTP involved the expansion of the coverage of a government funded non-contributory health insurance (Green Card scheme) and benefits package for uninsured population groups, namely the poor, which included low-income households whose earnings were below the minimum wage, and the unemployed. Starting with the introduction of the HTP in 2003, the number of beneficiaries rose from 2.5 million in 2003 to about 9 million in 2011 [13].

There are three important qualifications necessary to obtain a Green Card. First: the applicant should not be registered with any of the social security institutions existing in Turkey, namely Social Insurance Organization for blue-collar workers (SIO), Government Employees Retirement Fund (GERF) for retired civil servants, Active Civil Servants Insurance Fund for civil servants in work and their dependents, and Bağ-Kur, the social health insurance scheme for those working in the informal sector including the self-employed, artisans, merchants and agricultural workers. Second: the total reported household income divided by the number of people in the household must be less than one third of minimum wage. Third: even if the household satisfies the first two qualifications, a Green Card could be denied if the district committee administering the program determines that a household is not poor [14].

It is the second qualification that we exploit in our study to estimate the impact of having a Green Card on health service utilization. It is important to note that while the household monthly per capita income is utilized to determine the eligibility, the enrollment to the Green Card scheme is made individually.

In its early phases the HTP helped to improve access to maternal and child health services – particularly antenatal visits, delivery in a health facility, and immunization uptake – which contributed to significant declines in under-5 mortality, and infant mortality [2]. While some have questioned the impact of HTP on improving outcomes and reducing health inequalities, these assertions were not supported by robust evidence [15]. Further evidence suggests that the Green Card scheme protected beneficiaries from foregoing health care utilization during the recession experienced by Turkey in 2009, when Green Card holders reduced their use of services less than those who did not possess one [14]. However, the study did not account for the self-selection into the Green Card scheme and other key confounders, such as changes in income levels.

Our study extends earlier analyses on health care utilization and access by the poor following the expansion of Green Card coverage. We take advantage of a sharp break in the availability of Green Card for households whose monthly per capita income is at or below 243 Turkish Lira (TL), which is one third of the then minimum wage, to examine, for the first time, the impact of the Green Card scheme on the utilization of outpatient, inpatient, specialist, and diagnostics services by the poor.

We use a kinked regression design [16], which contrasts the rapid rate of decline in Green Card coverage at monthly income level of 243 TL (the cut off) with low but stable level of possession by those whose monthly income exceeds 243 TL. We compare the difference in the availability of health insurance coverage to the changes in utilization of health services by those with incomes above and below the cut-off. An abrupt shift in the relationship between income and utilization would indicate a likely causal relationship between Green Card possession and the utilization of services. Moreover, for our method to be valid, the smoothness condition indicates that the distribution of predetermined covariates around the kink point cannot show a kink at the cut-off.

## DATA AND METHODS

**Data** We use the complete data set from the nationally representative Turkey 2010 Health Survey undertaken by the Turkish Statistical Institute (TurkStat) [17]. The Turkey 2010 Health Survey provides information on household size, household monthly income in ranges and self-reports on the use of health care services by members of the household for individuals aged 15 years and older. The Survey samples all settlements in the territory of the Republic of Turkey. Institutional populations (soldiers, individuals living in dormitories, prisons, hospitals at the long terms, homes for the elderly, etc.) and small villages with population less than 132 persons are excluded from the survey [17]. In May-June 2010, 7886 households were surveyed to produce estimations for rural and urban areas, and for Turkey as a whole.

In the 2010 Health Survey data, we observe both family size and the income range in which a household’s income lies. Households’ income brackets consist of 10 various categories including “Less than 350 TL”, “351-500 TL”, ”501-620 TL”, ”621-750 TL”, ”751-900 TL”, ”901-1100 TL”, ”1101-1300 TL”, ”1301-1700 TL”, ”1701-2300 TL”,” Above 2301 TL”.

The Health Survey derives many indicators on health including health conditions of infants, children and adults and also the utilization of health services, satisfaction levels from these services, difficulties faced during daily activities, and smoking and alcohol consumption for individuals 15 years old or more [17]. Further information on the 2010 Health Survey data set and the sampling design can be found in Appendix S1 of **Online Supplementary Document(Online Supplementary Document)**.

### Methods

We estimate a household’s income based on the range in which its income falls and approximate household income as either being in the middle or at the bottom end of the range. The latter method is justified by the well-known tendency for people in the lower half of the income distribution to over-report their income in surveys (while those in the upper half tend to under-report their income) [18]. Both estimation methods provide similar results, and we report results using per capita income derived from the midpoint of the range (all results available from authors). We subtract one third of the minimum wage from per capita income to center the constructed income variable at the eligibility cut off for the Green Card.

Ideally, any household with a positive value on this variable would be ineligible for a Green Card while those with negative values would qualify. However, there are several reasons why we may still observe some people with Green Cards above the cutoff point: (i) It is possible that some people with incomes above the cut-off receive Green Cards by under-reporting their income (ii) People may not correctly report their household income in our survey (iii) People may not correctly report their Green Card status, (iv) Our estimate of household income is only approximate since we only know the range in which people’s income falls. Thus, we expect that some people with reported family incomes above the cut-off will report having a Green Card. Nonetheless, we do observe a rather abrupt change in the slope of the relationship between per capita income and possession of a Green Card at exactly one third of the minimum wage.

We implement the fuzzy regression kink method using the local linear regression design, as suggested by Hahn and others [19]. In this case we restrict the sample to values of our constructed variable in the neighborhood of zero (the cut off point for being eligible for a Green Card in our constructed variable). We first choose symmetric bandwidths, namely 150 TL away from 0 TL on each side of the cutoff for the income variable around the kink point centered at zero. A bandwidth of 150 TL allows for accurate estimates while keeping the estimates in a narrow range around the cut off (standard deviation of income is 325 TL and the largest symmetric bandwidth is 238 TL). Sample sizes were too small to provide accurate estimates of the slopes at bandwidths of 50 TL, and results at 100 TL and 200 TL were qualitatively similar to those reported here (Table S1-S2 in **Online Supplementary Document(Online Supplementary Document)**). 7729 individuals had per capita income between 93 TL and 393 TL. Individuals are categorized into various groups (‘bins’) based on their per capita family income. We let the software choose the number of ‘bins’ on each side of the cut off. For most of the figures there is insufficient data to show all ‘bins’ to the right of the income cut-off as the income levels increase).

A regression kink design (RKD) is a term that Nielsen and his colleagues conceived in a pioneering article [20]. This method carries very similar (almost the same) procedures in its implementation like a regression discontinuity design (RDD) except that in the former case (RKD) researchers take into consideration an abrupt kink in the relationship between the assignment variable (per capita income) and the policy variable (Health insurance for the poor in our case) [21]. However, in an RDD method we look for a discontinuity in the assignment variable to make a causal inference. In an RKD type of analysis, we investigate the relationship exactly where a kink (which we observe in our study right at one third of minimum wage) occurs in the slope of the relationship between the dependent variable and the assignment variable. The main assumption in an RKD method is to have “similar” individuals on either side of the cut off so that any kink that we observe in the slope of the outcome variable shall be accredited to the treatment impact of the policy variable of interest which is the health insurance program for the poor in Turkey in this study.

We then regress our utilization variables on a dummy variable for having income below the Green Card cutoff, the constructed variable reflecting per capita family income, and the interaction between these two variables in order to get the slope below the cut-off point. We compute the impact of having insurance by comparing the slopes of the regression above and below the cutoff. The local linear regression specifications [19] are as follows:

D_i_ = β_0_ + β_1_ T_i_ + β_2_ (inc_i_ – 243) + β_3_ (inc_i_ – 243) T_i_ +e_i_ (1)

Y_i_ = β_4_ + β_5_ T_i_ + β_6_ (inc_i_ – 243) + β_7_ (inc_i_ – 243) T_i_ +u_i_ (2)

where D is a binary indicator of Green Card status for individual i, Y is an outcome variable (outpatient, inpatient, specialist, and diagnostics services utilisation), T is a binary indicator of the per capita income less than 243 TL (one third of the minimum wage), and inc is per capita income in the household. β_0_ through β_7_ are coefficients to be estimated and e and u are error terms. Equations 1 and 2 are estimated on individual data by ordinary least squares using robust standard errors which are clustered at the household level.

We examine whether having a Green Card leads to increased utilization of health services by assuming that the relationship between income and health care utilization apparent to the right of the cut-off, where no one should have a Green Card, would continue to the left of the cut-off where individuals have Green Card [16,22,23]. The impact of having a Green Card on service utilization, ie, as regression kink design, is estimated by taking the ratio of the magnitude of the kinks in both the ‘treatment’ (having Green Card; β_3_) and the outcome variables (β_7_).

### Design validity

Causal imputation from regression discontinuity designs depends primarily on one important assumption; that the only difference between those on one side of the discontinuity and the other is the change in the probability of receiving the ‘treatment’ which in our case is having a Green Card [19]. This assumption of causal imputation is also important for the regression kink design.

It is possible to check the validity of this assumption by first showing a kink in the ‘treatment’ (having Green Card) at zero on our per capita income variable that is centered at the eligibility cut off for the Green Card, and then showing that the kink is not present in other characteristics of individuals or in the utilization of health services not covered by the Green Card. These variables should not show any statistically significant kink or discontinuity at the cut-off point for Green Card eligibility. We examine 41 variables (Table S3 in **Online Supplementary Document(Online Supplementary Document)**) for kinks at the cut off for Green Card eligibility. We find for these 41 variables fewer statistically significant breaks around the limit income for the Green Card than we would expect to find by chance. Having shown that it is unlikely that there is any significant discontinuity in the level or slope of potential covariates (Figure S1 and Table S3-S4 in **Online Supplementary Document(Online Supplementary Document)**) there is no reason to include these 41 variables in the regression design, and doing so does not impact our results significantly (Table S5 in **Online Supplementary Document(Online Supplementary Document)**).

Another key assumption in an RKD method is to test whether there is manipulation in the assignment variable (in this study self-reported household income). The main idea is to have a smooth density condition where the assignment variable is continuously differentiable at the cut off. We test this assumption by running a density test [24] on household per capita income which is our assignment variable in this study. As we have individuals as our units of analysis the test will face so many duplicates with the same income from the same household. This might create a potential problem for the test. Therefore, to do the smooth density test at the threshold, we delete duplicate observations based on income from each household and leave only one member of a household. The graph of the McCrary test can be found in Figure S2 in **Online Supplementary Document(Online Supplementary Document)** and the t-statistic for continuous slope at kink-point is 0.906.

## RESULTS

Descriptive statistics for Green Card enrollees (treated) and non-green card holders (comparison) indicate that average of the dependent variables is very similar for both groups (Table S6 in **Online Supplementary Document(Online Supplementary Document)**). Going down in the table we see that people without Green Card insurance are more likely to be in urban region and more educated. They are also more inclined to use tobacco and alcohol products. The rest of the control variables show similar results. These similarities with respect to some pre-determined characteristics along with other validity checks between treatment and comparison groups firmly state that individuals falling into these groups in the specified bandwidth (150 TL on both sides away from 0 TL) are comparable.

**Figure 1:**
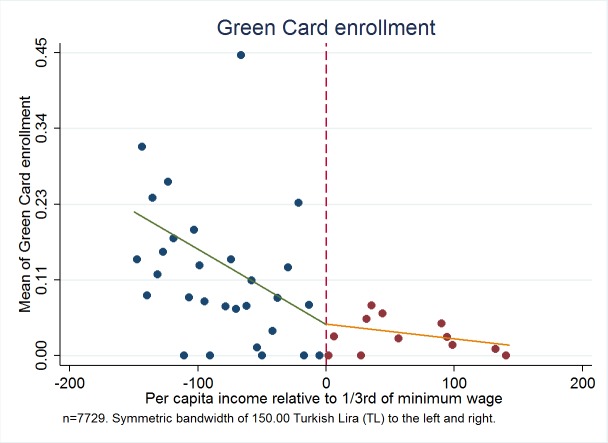
Fraction of green card holders for the bandwidth of 150.00 Turkish Lira (TL) to the left and right. Authors’ analysis, using data from Turkish Statistical Institute (TurkStat) Turkey 2010 Health Survey. Data Source: Turkey 2010 Health Survey Data from TurkStat are available by request.

**Figure 2:**
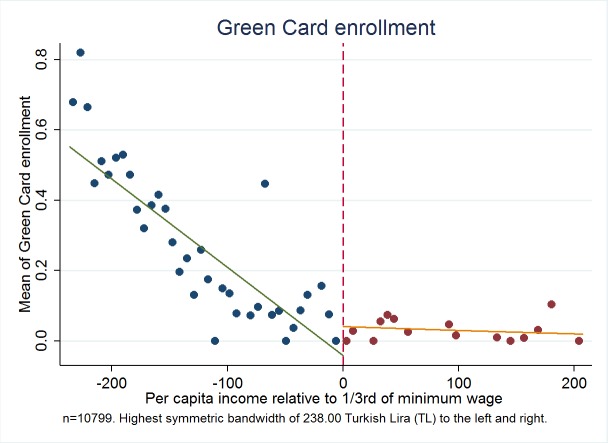
Fraction of green card holders for the bandwidth of 238.00 Turkish Lira (TL) to the left and right. Authors’ analysis, using data from Turkish Statistical Institute (TurkStat) Turkey 2010 Health Survey. Data Source: Turkey 2010 Health Survey Data from TurkStat are available by request.

**Figure 1** illustrates the relationship between centered per capita income and the probability of having a Green Card around the kink point centered at zero. Note that in this and the rest of the figures, we utilize a range of ±150.00 TL. The fraction of people with a Green Card in each range is shown on the vertical axis at the center of the corresponding income range, which is displayed on the horizontal axis. A similar arrangement is used in the other figures in this paper where the vertical axis represents use of services or other outcomes. **Figure 2** shows this relationship for a range of ±238.00 TL below and above the cut-off point, as this is the largest symmetric bandwidth around the cut-off kink that we can report in our data.

**Figure 1** shows a very clear kink in the relationship between per capita income in the household and the probability of having Green Card. Above the cut-off, as income increases, the probability of having Green Card falls, with a change in slope as per person income pass through the threshold after which the probability of having a Green Card remains virtually constant at a very low level. Moreover, **Figure 2** shows clearer and less dispersed relationship between income and the probability of having a Green Card health insurance, where we use the largest symmetric range around the kink point.

**Figure 3:**
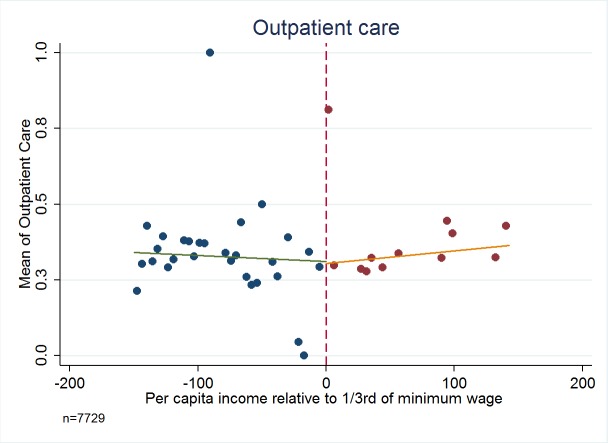
Outpatient visit – Have you been to outpatient services in last 12 months? Dependent variables are binary values, taking a value of 1 if the incident (in relation to each question) has been realized, and 0 if the incident has not been realized. Authors’ analysis, using data from Turkish Statistical Institute (TurkStat) Turkey 2010 Health Survey. Data Source: Turkey 2010 Health Survey Data from TurkStat are available by request.

**Figure 4:**
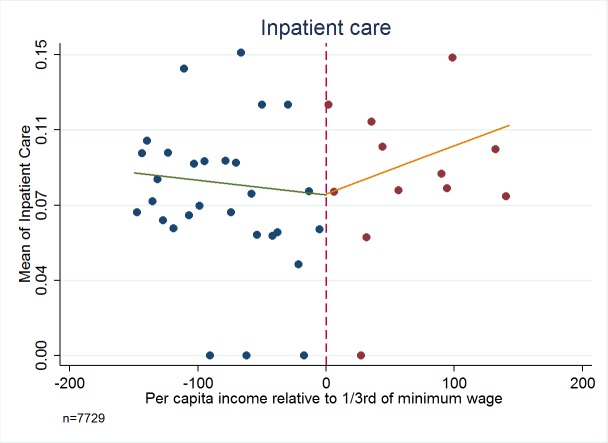
Inpatient visit – Have you been to inpatient services in last 12 months? Dependent variables are binary values, taking a value of 1 if the incident (in relation to each question) has been realized, and 0 if the incident has not been realized. Authors’ analysis, using data from Turkish Statistical Institute (TurkStat) Turkey 2010 Health Survey. Data Source: Turkey 2010 Health Survey Data from TurkStat are available by request.

**Figure 5:**
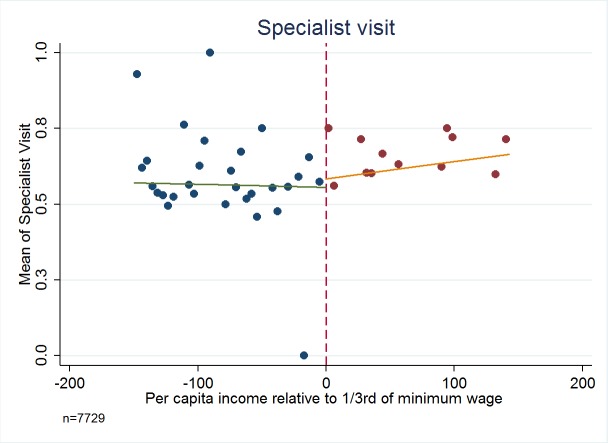
Specialist visit – When was the last time you visited a specialist? Dependent variable is a categorical variable indexed for shorter than 12 months (coded as 1), longer than 12 months (coded as 0) including “Never” option. Authors’ analysis, using data from Turkish Statistical Institute (TurkStat) Turkey 2010 Health Survey. Data Source: Turkey 2010 Health Survey Data from TurkStat are available by request.

**Figure 6:**
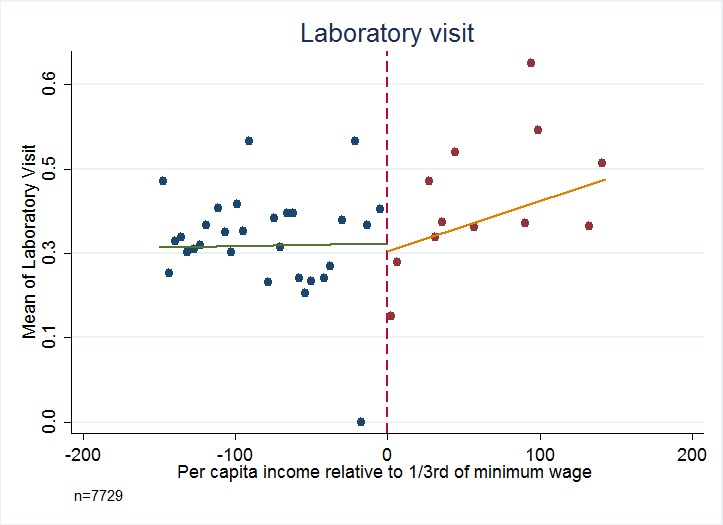
Diagnostic services: Laboratory visit – Have you been to laboratory services in last 12 months? Dependent variables are binary values, taking a value of 1 if the incident (in relation to each question) has been realized, and 0 if the incident has not been realized. Authors’ analysis, using data from Turkish Statistical Institute (TurkStat) Turkey 2010 Health Survey. Data Source: Turkey 2010 Health Survey Data from TurkStat are available by request.

**Figures 3** to **6** present the relationship between the service utilization outcome variables examined (**Figure 3** outpatient, **Figure 4** inpatient, **Figure 5** visit to a specialist, and **Figure 6** laboratory diagnostic services) and per capita income in the household. Kinks in utilization at the cut-off point are present in all cases, with service utilization increasing progressively for the poor population with Green Card coverage (ie, poorer groups utilise more). **Figures 3** to **6** also show that for those without Green Card rising level of utilization with increasing income levels.

**Tables 1** and** 2** present estimates of kinks in the proportion of people possessing Green Cards, and the fraction of people utilizing health services respectively. **Table 2** shows that possession of Green Card is progressive and significantly benefits the poor (*P* < 0.01). For example, last column of **Table 2** indicates that for every 100 TL increase in per capita income there will be about 9 percentage points decline in possessing a Green Card health insurance. For health care utilization variables of outpatient, inpatient, specialist consultation and laboratory service use, in every case the variables examined show a statistically significant kink at the cut-off for Green Card eligibility.

**Table 1:** Local linear regression of possession of Green Card on income*

	Constant (β_0_)	Difference in intercepts (β_1_)	Slope above break point (β_2_)	Difference in slopes above and below break point (β_3_)
Had Green Card	0.0469† (0.0083)	0.0000 (0.0166)	-0.0002‡ (0.0001)	-0.0899† (0.0221)

*Authors’ analysis of data from Turkish Statistical Institute (TurkStat) Turkey Health Survey 2010. Dependent variables are binary values, taking a value of 1 if the incident (possession of Green Card) has been realized, and 0 if the incident has not been realized. Independent variable income is measured in hundreds of Turkish Lira (TL) above or below 1/3rd of minimum wage (243.00 TL per month). The results are for estimation with 150.00 TL bandwidth. N = 7729.

†*P* < 0.01

‡*P* < 0.05.

**Table 2:** Local linear regression estimates of health care utilization outcomes for incomes around a bandwidth of 150.00 Turkish Lira above or below minimum wage (break point) of 243.00 Turkish Lira*

Question in household survey	Constant (β_4_)	Difference in intercepts (β_5_)	Slope above break point (β_6_)	Difference in slopes above and below break point (β_7_)	Estimate of effect (β_7_/β_3_)
Outpatient: Have you been to outpatient services in last 12 mo?	0.3065† (0.0212)	0.0057 (0.0286)	0.0004 (0.0003)	-0.0614§ (0.0344)	0.6830‡ (0.3021)
Inpatient: Have you been to inpatient services in last 12 mo?	0.0807† (0.0095)	0.0004 (0.0132)	0.0002‡ (0.0001)	-0.0311§ (0.0163)	0.3459§ (0.1786)
Special: When was the last time you visited a specialist?	0.5890† (0.0191)	-0.0297 (0.0267)	0.0005‡ (0.0002)	-0.0666‡ (0.0313)	0.7408‡ (0.3134)
Laboratory: Have you been to laboratory in last 12 mo?	0.3032† (0.0179)	0.0154 (0.0248)	0.0009† (0.0002)	-0.0863† (0.0303)	0.9600† (0.3227)

*Authors’ analysis of data from Turkish Statistical Institute (TurkStat) Turkey Health Survey 2010. Dependent variables are binary values, taking a value of 1 if the incident (in relation to each question) has been realized, and 0 if the incident has not been realized except for “Special-When was the last time you visited a specialist?” for which the dependent variable is a categorical variable indexed for shorter than 12 mo (coded as 1), longer than 12 mo and ‘Never’ (coded as 0). Utilization data are self-reports on the use of health care services by members of the household for individuals 15 y and older. Independent variable income is measured in hundreds of Turkish Lira (TL) above or below 1/3rd of minimum wage (243.00 TL). The results are for estimation with 150.00 TL bandwidth. N = 7729.

†*P* < 0.01.

‡*P* < 0.05.

§*P* < 0.10.

Analysis using the standard regression kink method yields very large impacts of Green Card possession on use of health care services. For example, having a Green Card increases the fraction of people using outpatient services by 68.30 percentage points (*P* = 0.024, 95% confidence interval CI = 67.6-69.0), inpatient visit by 34.60 percentage points (*P* = 0.052, 95% CI = 34.2-35.0), and specialist visit by 74.10 percentage points (*P* < 0.019, 95% CI = 73.4-75.0). The point estimate for the impact of having a Green Card on using laboratory services at least once during the last year was to increase the probability by 96.00 percentage points (*P* < 0.003, 95% CI = 95.3-96.7) and in all cases confidence intervals include plausible values.

## DISCUSSION

In this study, we use regression kink design, a strong quasi-experimental study method, to assess the impact of expanded access to health insurance coverage and benefits for the poor through the Green Card scheme in Turkey on health care utilization. We find very large and statistically significant effects of Green Card possession on the utilization of a wide range of health care services which were included in the expanded benefits package for Green Card beneficiaries, namely outpatient, inpatient and laboratory diagnostic services, as well as consultation with specialists. This is the first time regression kink design has been used to examine the effects of expansion of health insurance coverage for the poor on their utilization of health services.

Earlier studies, which have analysed HTP in Turkey, have shown contradictory results. One recent study indicates that the health system reforms brought by HTP in Turkey have failed to reach the poor in the sense that the large portion of the poor still lack necessary public insurance coverage [25]. However, another study report there has been a significant progress in the health status of the Turkish population and an improvement in performance indicators with the introduction of health reforms [26]. Further analysis of HTP revealed socioeconomic determinants of inequalities in the use of health care services by adults and suggested that there were inequalities in some of the health care services, which is related to income, education, and geographical region [27]. Our results, which uses regression kink design, a strong quasi-experimental method, provide a convincing case for causal relationship, and produce larger estimates of the impact than are typical elsewhere in the literature.

Our results also suggest a causal impact of expanding UHC coverage on improving health care utilization for the poor. Analysis of causal impact of policies is complicated by the fact that individuals can choose whether to take part based on their expectations of the policies’ effects for them. Regression discontinuity and regression kink designs are non-experimental methods that allow convincing estimates of causal relationships. When there is a policy variable (determined by a formula) which is based on an endogenous assignment variable (in our case one-third of minimum wage), conventional estimation methods will not work in these settings [16]. An RKD method provides a better way to deal with these sorts of implementations when there is a policy function which is kinked at a certain threshold [20].

Our finding of statistically significant effect of the Green Card scheme on utilization of health services hinges critically on the assumption that except for Green Card eligibility, other characteristics of people that might influence health care coverage change smoothly and at a constant rate across the break point for family eligibility for a Green Card. The plausibility of this assumption is supported by our inability to find evidence against this assumption in our analysis of 41 variables (82 coefficients), and by the fact that services not covered by the Green Card show no kink at the income cut-off. However, only random assignment to treatment and control conditions could assure us of a lack of systematic bias. When introducing large-scale nationwide policy change, such experiments would face daunting political, procedural, ethical and scale problems to randomly allocate citizens or geographic areas to treatment and no treatment, and in health few examples of large-scale randomized controlled studies exist [8,28].

Another limitation of our method is that to estimate the magnitude of treatment effects we have to assume that the relationship between income and utilization of services would be linear throughout the entire, though limited, range of income levels we study.

Notwithstanding limitations, our study has shown a large and statistically significant effect of the Green Card scheme on the utilisation of health services by the poor following major expansion of insurance coverage and the benefits package. Our findings suggest that non-contributory health insurance program, like Turkey’s Green Card scheme, could help significantly increase access to a number of health care services by the poor. Experience of Turkey is highly relevant for low- and middle-income countries, especially those transitioning to UHC, which aim to introduce new health programs targeting the poor.

It would also be beneficial to shed some light on advantages of a non-contributory scheme. Green card is for families who do not have capacity to pay. However, families who are able to contribute financially do so through compulsory social insurance scheme which includes Social Insurance Organization for blue-collar workers (SIO), Government Employees Retirement Fund (GERF) for retired civil servants, Active Civil Servants Insurance Fund for civil servants in work and their dependents, and Bağ-Kur, the social health insurance scheme for those working in the informal sector including the self-employed, artisans, merchants and agricultural workers. The contribution by families enables solidarity where better-off (families with capacity to pay) subsidize those who are not able to do so. In the case of Turkey, two factors made Green Card possible. One is large risk pool by unified scheme, second progressive nature of contribution by those who can pay, which enables achievement of solidarity.

Our study provides an evidence in one dimension of potential benefits of UHC in the presence of a non-contributory scheme namely Green Card insurance. In order to show full benefits of UHC it would be important to explore other dimensions such as improvement in health outcomes, financial protection, and user satisfaction [2].

Our study shows the benefits of UHC with large-scale non-contributory schemes enabled by creating a large risk pool and solidarity. Further, our findings indicate that it is possible to level up health insurance benefits to eliminate a two-tier system so that there is no discrimination of the poor whose benefits realized through a non-contributory health insurance scheme. Furthermore, our study indicates that the poor are able to realize benefits of health insurance and are able to increase utilization of health care services in the presence of a sound benefits package and supply side measures.
