# Supplementary material for: Early experience of universal health coverage in Turkey on access to health services for the poor: regression kink design analysis
Source: J Glob Health. 2018 Sep 19;8(2):020412. doi: 10.7189/jogh.08.020412 (PMC6150612; doi:10.7189/jogh.08.020412)
Supplement: Online Supplementary Document [file jogh-08-020412-s001.pdf]

Online Supplementary Document

Tirgil et al. Early experience of universal health coverage in Turkey on access to health services for the poor: regression kink design analysis

J Glob Health 2018;8:020412

Some control variables used for covariates balance check:

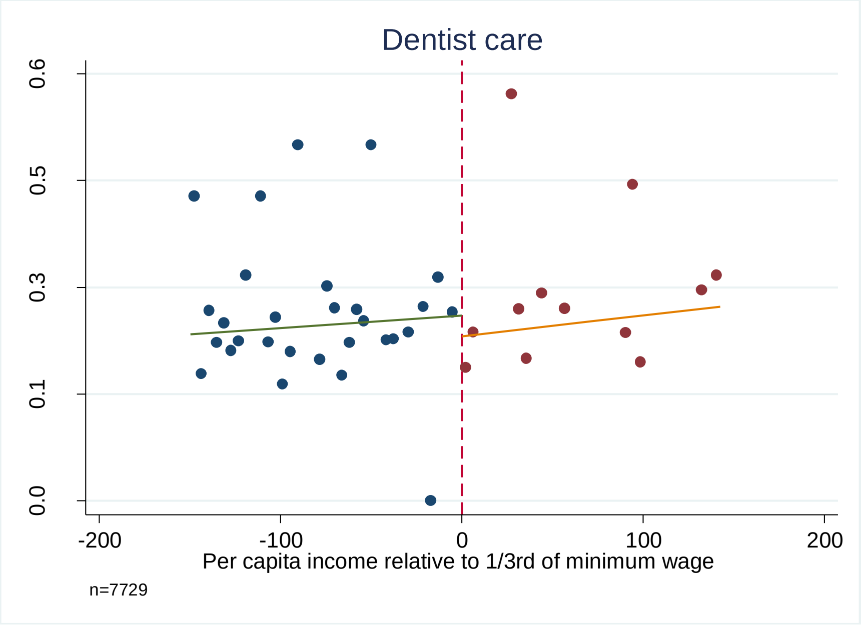

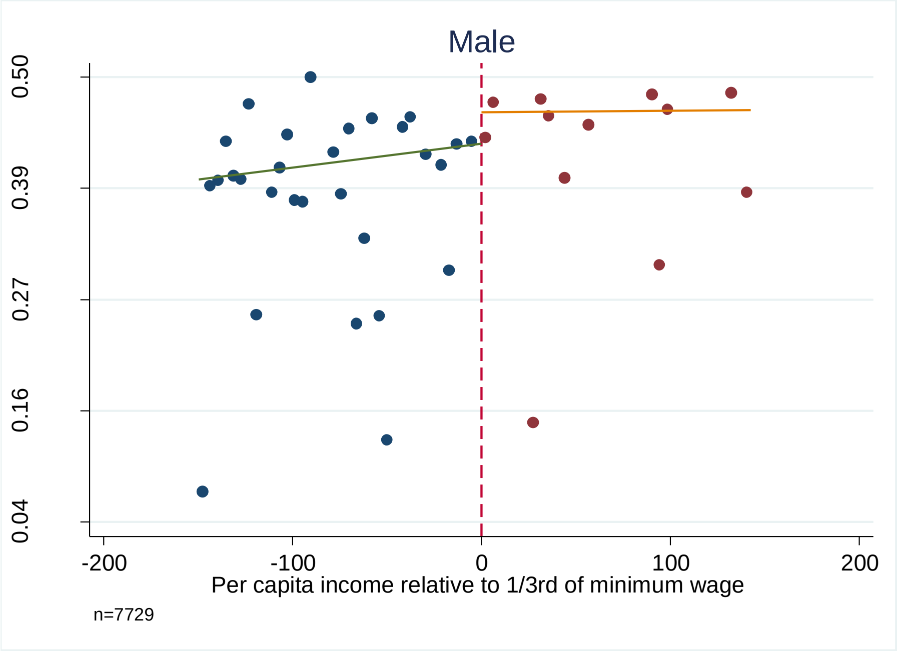

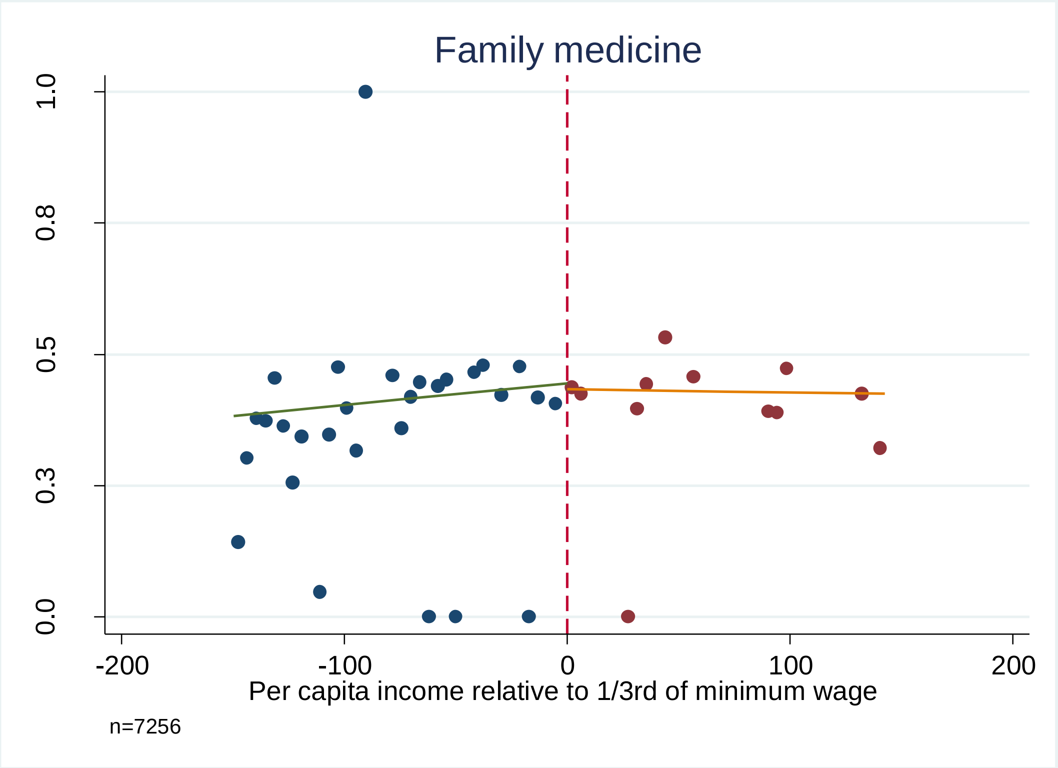

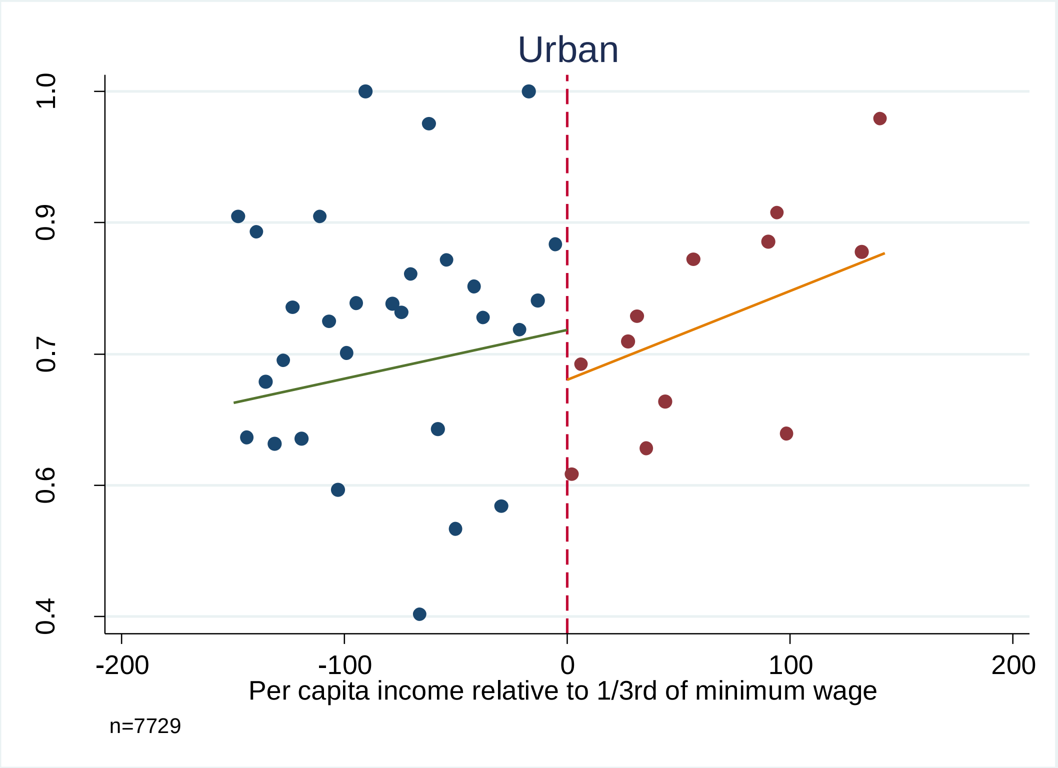

Figure S1: Fraction of four main covariates around the cut off income level

(A) Dentist visit - When was the last time you visited dentist? (B) Male. (C) Existence of family medicine program (D) Urban - Where do you live in? Authors' analysis, using data from Turkish Statistical Institute (TurkStat) Turkey 2010 Health Survey.

Data Source: Turkey 2010 Health Survey Data from TurkStat are available by request.

McCrary density test:

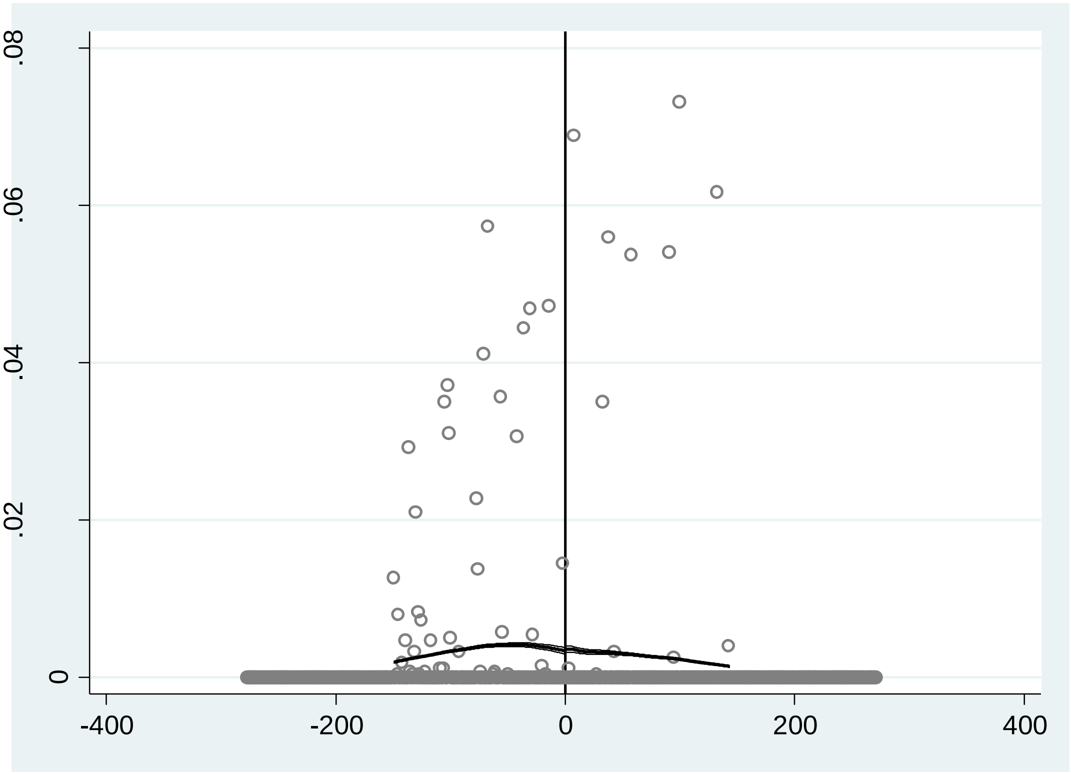

Figure S2: Density at the cut off income.

X axis is the “per capita income relative to 1/3rd of minimum wage”. Y axis is the frequency. Authors’ analysis, using data from Turkish Statistical Institute (TurkStat) Turkey 2010 Health Survey.

Data Source: Turkey 2010 Health Survey Data from TurkStat are available by request.

Question in Household Survey	Constant	Difference in intercepts	Slope above break point	Difference in slopes above and below break point	Estimate of Effect
Had Green Card	0.0419**<br>(0.0092)	0.0084<br>(0.0197)	-0.0000<br>(0.0001)	-0.1010*<br>(0.0412)	-
Outpatient-Have you been to outpatient services in last 12 months?	0.2933**<br>(0.0236)	0.0089<br>(0.0355)	0.0007*<br>(0.0004)	-0.1094+<br>(0.0620)	1.0832*<br>(0.5247)
Inpatient-Have you been to inpatient services in last 12 months?	0.0737**<br>(0.0104)	-0.0008<br>(0.0160)	0.0004*<br>(0.0002)	-0.0687*<br>(0.0288)	0.6802*<br>(0.3213)
Special-When was the last time you visited a specialist?	0.5579**<br>(0.0211)	0.0060<br>(0.0336)	0.0013**<br>(0.0003)	-0.1239*<br>(0.0579)	1.2257*<br>(0.5677)
Laboratory-Have you been to lab in last 12 months?	0.2694<br>(0.0195)	0.0688*<br>(0.0310)	0.0018**<br>(0.0003)	-0.1248<br>(0.0566)	1.2356*<br>(0.5565)

Authors' analysis of data from Turkish Statistical Institute (TurkStat) Turkey Health Survey 2010. Dependent variables are binary values, taking a value of 1 if the incident (in relation to each question) has been realized, and 0 if the incident has not been realized except for "Special-When was the last time you visited a specialist?" for which the dependent variable is a categorical variable indexed for shorter than 12 months (coded as 1), longer than 12 months (coded as 0). Utilization data are self-reports on the use of health care services by members of the household for individuals 15 years and older. Independent variable income is measured in hundreds of Turkish Lira (TL) above or below 1/3rd of minimum wage (243·00 TL). The results are for estimation with 100·00 TL bandwidth. **p<0·01. *p<0·05. +p<0·10. N = 5276.

Table S1: Local Linear Regression Estimates of Health Care Utilization Outcomes for incomes around a bandwidth of 100·00 Turkish Lira above or below minimum wage (break point) of 243·00 Turkish Lira

Question in Household Survey	Constant	Difference in intercepts	Slope above break point	Difference in slopes above and below break point	Estimate of Effect
Had Green Card	0.0405** (0.0078)	-0.0648** (0.0156)	-0.0001 (0.0001)	-0.2192** (0.0166)	-
Outpatient-Have you been to outpatient services in last 12 months?	0.3137** (0.0189)	-0.0086 (0.0250)	0.0002 (0.0002)	-0.0544* (0.0230)	0.2482** (0.080)
Inpatient-Have you been to inpatient services in last 12 months?	0.0830** (0.0087)	-0.0053 (0.0118)	0.0002* (0.0001)	-0.0304** (0.0111)	0.1387** (0.049)
Special-When was the last time you visited a specialist?	0.5965** (0.0172)	-0.0404+ (0.0234)	0.0003+ (0.0002)	-0.0389+ (0.0212)	0.1775* (0.082)
Laboratory-Have you been to lab in last 12 months?	0.3201** (0.0164)	0.0056 (0.0220)	0.0005** (0.0002)	-0.0364+ (0.0206)	0.1661* (0.079)

Authors' analysis of data from Turkish Statistical Institute (TurkStat) Turkey Health Survey 2010. Dependent variables are binary values, taking a value of 1 if the incident (in relation to each question) has been realized, and 0 if the incident has not been realized except for "Special-When was the last time you visited a specialist?" for which the dependent variable is a categorical variable indexed for shorter than 12 months (coded as 1), longer than 12 months (coded as 0). Utilization data are self-reports on the use of health care services by members of the household for individuals 15 years and older. Independent variable income is measured in hundreds of Turkish Lira (TL) above or below 1/3rd of minimum wage (243·00 TL). The results are for estimation with 200·00 TL bandwidth. **p<0·01. *p<0·05. +p<0·10. N = 10098.

Table S2: Local Linear Regression Estimates of Health Care Utilization Outcomes for incomes around a bandwidth of 200·00 Turkish Lira above or below minimum wage (break point) of 243·00 Turkish Lira

Dependent variable	Difference in intercepts	Difference in slopes above and below break point
**Family medicine exists**	0·0106<br>(0·0387)	0·0004<br>(0·0004)
**Education in years**	-0·0570<br>(0·0562)	-0·0005<br>(0·0007)
**Urban**	0·0565<br>(0·0351)	-0·0005<br>(0·0004)
**Family medicine visit**	-0·0026<br>(0·0277)	0·0000<br>(0·0003)
**Dentist**	0·0288<br>(0·0218)	-0·0001<br>(0·0003)
**Male**	-0·0321*<br>(0·0145)	0·0002<br>(0·0002)
**Employment Status**	-0·0054<br>(0·0204)	-0·0000<br>(0·0002)
**Ever used tobacco**	-0·0143<br>(0·0237)	-0·0001<br>(0·0002)
**Ever Used Alcohol**	-0·0069<br>(0·0216)	0·0000<br>(0·0002)
**Domestic Violence**	-0·0029<br>(0·0095)	0·0000<br>(0·0001)
**Exposure to Noise at Home (Traffic)**	-0·0011<br>(0·0259)	0·0001<br>(0·0003)
**Exposure to Air Pollution at Home**	-0·0074<br>(0·0256)	-0·0002<br>(0·0003)
**Exposure to Bad Scent at Home**	0·0447<br>(0·0258)	0·0000<br>(0·0003)
**Job Leave due to Health Problems**	0·0255<br>(0·0275)	0·0001<br>(0·0003)
**Fall Sick due to Job**	0·0030<br>(0·0126)	0·0001<br>(0·0001)

Authors' analysis of data from Turkish Statistical Institute (TurkStat) Turkey Health Survey 2010. Institute TS. Health Survey. In: Institute TS, editor. Ankara: Turkish Statistical Institute; 2010. Dependent variables are binary values, taking a value of 1 if the incident has been realized, and 0 if the incident has not been realized. Data are self-reports by members of the household for individuals 15 years and older. The results are for estimation with 150·00TL bandwidth above or below minimum wage. **p<0·01. *p<0·05. +p<0·10.

Table S3: Local Linear Regression Estimates of Covariates

Dependent variable	Difference in intercepts	Difference in slopes above and below break point
**Asthma**	-0·0009<br>(0·0102)	0·0001<br>(0·0001)
**Thyroid**	0·0049<br>(0·0081)	0·0001<br>(0·0001)
**Bronchitis**	-0·0074<br>(0·0102)	-0.0002<br>(0.0001)
**Chronic Hepatitis**	0·0011<br>(0·0063)	0·0001<br>(0·0001)
**Spasm**	0·0076<br>(0·0106)	-0·0001<br>(0·0001)
**Heart Failure**	0·0137*<br>(0·0064)	-0·0001^{+}<br>(0·0001)
**Chronic Hypertension**	-0·0039<br>(0·0161)	0·0002<br>(0·0002)
**Cerebral Hemorrhage**	0·0019<br>(0·0047)	0·0000<br>(0·0001)
**Calcification**	0·0005<br>(0·0146)	0·0001<br>(0·0002)
**Rheumatism**	0·0103<br>(0·0159)	-0·0001<br>(0·0002)
**Backache**	0·0204<br>(0·0184)	-0·0002<br>(0·0002)
**Cervical Disk Problems**	0·0231<br>(0·0138)	-0·0002<br>(0·0002)
**Chronic Diabetes**	-0·0086<br>(0·0111)	0·0001<br>(0·0001)
**Allergy**	0·0062<br>(0·0119)	-0·0001<br>(0·0001)
**Gastric Ulcer**	0·0023<br>(0·0143)	0·0001<br>(0·0002)
**Duodenal Ulcer**	0·0058<br>(0·0053)	-0·0001<br>(0·0001)
**Liver Cirrhosis**	0·0010<br>(0·0038)	0·0000<br>(0·0001)
**Cancer**	0·0025<br>(0·0028)	0·0000<br>(0·0000)
**Migraine**	-0·0054<br>(0·0127)	-0·0001<br>(0·0002)
**Urinary**	0·0052<br>(0·0093)	-0·0002<br>(0·0001)
**Chronic Anxiety**	-0·0006<br>(0·0052)	-0·0001<br>(0·0001)
**Chronic Depression**	-0·0054<br>(0·0083)	-0·0001<br>(0·0001)
**Mental Illness**	0·0082<br>(0·0065)	-0·0001<br>(0·0001)
**Anemia**	-0·0083<br>(0·0135)	-0·0000<br>(0·0002)
**Continuous Injury**	0·0007<br>(0·0056)	0·0001<br>(0·0001)
**Sinus**	0·0129<br>(0·0127)	0·0002<br>(0·0002)

Authors' analysis of data from Turkish Statistical Institute (TurkStat) Turkey Health Survey 2010. Institute TS. Health Survey. In: Institute TS, editor. Ankara: Turkish Statistical Institute; 2010. Dependent variables are binary values, taking a value of 1 if the incident has been realized, and 0 if the incident has not been realized. Data are self-reports by members of the household for individuals 15 years and older. The results are for estimation with 150·00TL bandwidth above or below minimum wage. **p<0·01. *p<0·05. +p<0·10.

Table S3 Cont'd: Local Linear Regression Estimates of Covariates

Correspondent questions regarding control variables asked in the survey:

Covariates	Explanation
**Dentist visit**	When was the last time you visited a dentist? - Shorter than 12 months (coded as 1), Longer than 12 months (coded as 0)
**Family Medicine exists**	Dummy for existence of family medicine program in the area
**Education in years**	Highest grade completed
**Male**	Dummy for male
**Urban**	Dummy for living in urban
**Ever used tobacco**	Have you ever used any tobacco products?
**Ever Used Alcohol**	Have you ever used any alcohol products?
**Domestic Violence**	Were you exposed to domestic violence in the last 12 months?
**Exposure to Noise at Home**	To what extent were you exposed to noise (traffic, rail traffic, air traffic, factories, neighbors, animals, restaurants, bars, and night clubs) at home in the last 12 months?
**Exposure to Air Pollution at Home**	To what extent were you exposed to air pollution (fine dust, scum, dust, smoke, and ozone) at home in the last 12 months?
**Exposure to Bad Scent at Home**	To what extent were you exposed to bad scent (industrial, agricultural, from a sewer, and from a trash) at home in the last 12 months?
**Employment Status**	Have you worked for pay in last 7 days?
**Job Leave due to Health Problems**	Have you taken a leave from your job in last 12 months due to health problems?
**Fall Sick due to Job**	Have you fallen ill in last 12 months due to your last or current job?
**Family Medicine visit**	Have you visited family medicine doctor in the last 12 months?

Table S4: Explanation of Some Covariates

Question in Household Survey	Constant	Difference in intercepts	Slope above break point	Difference in slopes above and below break point	Estimate of Effect
Had Green Card	0.2386** (0.0332)	-0.0052 (0.0164)	-0.000 (0.000)	-0.0996** (0.0210)	-
Outpatient-Have you been to outpatient services in last 12 months?	0.3776** (0.0557)	0·0049 (0·0283)	0.000+ (0.000)	-0.0684* (0.0347)	0.6867* (0.2740)
Inpatient-Have you been to inpatient services in last 12 months?	0.1932** (0.0413)	-0.0023 (0.0130)	0.000+ (0.000)	-0.0382* (0.0162)	0.3835* (0.1629)
Special-When was the last time you visited a specialist?	0.6339** (0.0466)	-0.0303 (0.0267)	0.001* (0.000)	-0.0658* (0.0318)	0.6606* (0.2778)
Laboratory-Have you been to lab in last 12 months?	0.3973** (0.0518)	0.0120 (0.0244)	0.001** (0.000)	-0.0864** (0.0300)	0.8675** (0.2812)

Authors' analysis of data from Turkish Statistical Institute (TurkStat) Turkey Health Survey 2010. Dependent variables are binary values, taking a value of 1 if the incident (in relation to each question) has been realized, and 0 if the incident has not been realized except for "Special-When was the last time you visited a specialist?" for which the dependent variable is a categorical variable indexed for shorter than 12 months (coded as 1), longer than 12 months (coded as 0). Utilization data are self-reports on the use of health care services by members of the household for individuals 15 years and older. Independent variable income is measured in hundreds of Turkish Lira (TL) above or below 1/3rd of minimum wage (243·00 TL). The results are for estimation with 150·00 TL bandwidth. **p<0·01. *p<0·05. +p<0·10. We include control variables in all regressions including urban, male, household size, marital status, educational level in groups, age in groups, heart failure status, ever used tobacco and alcohol status. N = 7729.

Table S5: Local Linear Regression Estimates of Health Care Utilization Outcomes for incomes around a bandwidth of 150·00 Turkish Lira above or below minimum wage (break point) of 243·00 Turkish Lira—Covariate adjusted

	Green Card sample (Treatment)			Non-Green Card sample (Comparison)	
	Mean	Std. Dev.		Mean	Std. Dev.
Outcome variables:					
**Outpatient**	0.35	0.48		0.33	0.47
**Inpatient**	0.12	0.33		0.09	0.28
**Specialist visit**	0.61	0.49		0.59	0.49
**Laboratory**	0.31	0.46		0.34	0.47
Control variables:					
**Urban**	0.52	0.50		0.73	0.45
**Male**	0.41	0.49		0.44	0.50
**Household size**	4.64	2.82		4.62	2.29
**Education in years**	3.82	3.97		6.46	4.20
**Married**	1.92	0.68		1.86	0.56
**Had heart failure**	0.02	0.13		0.02	0.14
**Ever used tobacco**	0.37	0.48		0.44	0.50
**Ever used alcohol**	0.15	0.36		0.22	0.42
**Number observations**	716			7013	

Notes: Authors' analysis of data from Turkish Statistical Institute (TurkStat) Turkey Health Survey 2010. We report means and standard deviations of outcome and control variables for treatment and comparison samples in the range of 150 TL on both sides of the cut off (0 TL). We also control for 'Age' which is listed as 7 categories in our regressions. We include "heart failure" in the analysis because it gives a statistically significant result when we do the covariates balance check in the design validity section.

Table S6. Summary Statistics for Treatment and Comparison Groups

Appendix S1. The information below is taken from the Turkish Statistical Institute (TurkStat) Turkey Health Survey 2010.

Purpose

The aim of the 2010 Health Survey is to introduce the health profile of individuals and to get information about health indicators which constitutes a big part of the development indicators that shows the degree of development of the countries. This survey has the specialty that held out to reflect the country in general and being comparable internationally (Turkish Statistical Institute Health Survey, 2010).

Estimation Level and Sample Size

The survey is designed in order to produce estimators for total of Turkey, urban and rural. Thus, the total sample size necessary was found to be 7 886 households.

Sampling Method

Strata and two-phase cluster sampling method was used0· For external stratification rural – urban difference was used. First phase sampling unit were blocks consisting of an average of 100 households and second phase sampling unit were households selected systematically from each block.

Address Frame

The frame used in the sampling of the survey was the "National Address Database" (NADB) which constitutes a base for "Address Based Registry System" (ABRS) which was completed in 2007. (Address frame was composed from NADB in March 2010). Settlements with population less than 133 were not included in the frame because it was considered that adequate number of sample households would not be reached.

Stratification Criterion

Settlements with population 20000 and below defined as “rural”, settlements with population 20001 and over defined as “urban”.

Sampling Distribution

In urban settlements; 5696 households were selected from 356 blocks which were previously selected (15 households in each block). In 4682 of these households the questionnaire was completed. In rural settlements; 2190 households were selected from 219 blocks which were previously selected (10 households from each block). In 1869 of these households the questionnaire was completed.

Weighting

Weighting procedures were carried out to obtain parameters from the data set resulting from sampling so as to represent the universe.

Firstly, base weights which are inversely proportional to the overall selection probabilities were calculated for each sample respondent. In this stage, it was calculated probabilities of selection of clusters, selection of households and then base weights were calculated by using these probabilities based on household and individual.

In the second stage of weighting, base weights were adjusted to compensate the losses in the sample outcome due to non-response.

In the final stage the weights are calibrated to the projected population distributions, using integrated calibration ratio method. The calibration is iterated and trimmed until any relative changes in the weights are within a pre-specified range. The final weights are then inflated by an overall inflation factor.
